# Outpatient Management of Diabetic Hand Infections

**DOI:** 10.7759/cureus.14263

**Published:** 2021-04-02

**Authors:** Ryan Qasawa, Daniel Yoho, Jenna Luker, Jake Markovicz, Aamir Siddiqui

**Affiliations:** 1 Surgery, Henry Ford Health System, Detroit, USA

**Keywords:** diabetes, hand, infection, surgery

## Abstract

Purpose

For many providers, hand infections among diabetic patients is a condition that necessitates focused inpatient care. These patients are believed to have decreased innate immunity to fight infection, a more virulent course, and difficulty with recovery. Diabetes is considered by some to represent an additional risk factor that can result in an unfavorable outcome if not managed in an aggressive manner. Our own experience suggests that many of these patients can be safely managed in the outpatient setting. The purpose of this project was to better define the clinical outcomes for this population.

Methods

Evidence-based criteria were utilized to direct inpatient versus outpatient treatment pathways. A database was developed to track hand infections treated by the specialty service. The primary outcome was the resolution of hand infection. Secondary outcomes included specific treatment responses as well as patient characteristic comparisons of the different treatment groups. Independent variables included (parenteral and enteral) antibiotic use and bedside interventions performed. Patients were followed to complete the resolution of infection.

Results

For all patients managed as outpatients, diabetic patients had statistically significantly decreased improvement rates at two weeks as compared to non-diabetic patients (62% vs 75%, p =0.024). This difference disappeared at two months. Among diabetic patients, those with the highest rate of recovery at two weeks (90%) received intravenous antibiotics, bedside procedures, and oral antibiotics. Patients who did not receive antibiotics or undergo bedside procedures had the lowest percent of improvement (37%). Across all treatment subgroups, bedside procedure was the most impactful intervention. Less than 10% of patients were converted from outpatient to inpatient care, both diabetic and non-diabetic.

Conclusions

We reviewed our experience managing diabetes mellitus hand infections treated in the outpatient setting. Appropriate and effective treatment is possible, and the results are equivalent to those of patients without diabetes mellitus.

## Introduction

Hand infections represent the majority of emergency department consultations for hand surgeons in large centers [1]. They run the spectrum from cellulitis to necrotizing soft tissue infections. Timely, effective, and evidence-based care are essential regardless of diagnosis. Hand infections receiving delayed, inappropriate, or ineffective treatment can result in permanent function impairment [2]. Increasingly, there is pressure to justify or minimize inpatient care. Admittedly, most treatment options, including exploration and debridement, wound care, antimicrobial therapy, and monitored surveillance, can be accomplished without an inpatient stay [3-7]. Success is predicated on identifying patients who can be treated safely, appropriately, and effectively in the outpatient setting.

Diabetes mellitus (DM) adds an extra dimension to the problem of hand infections [8-9]. Even when DM is controlled, these individuals can be at increased risk of ineffective host response, impaired healing, and delayed return of function [10-13]. For many hand surgeons, DM may represent an additional barrier to outpatient management because of concerns for a poor outcome or the need to convert to inpatient care. They may feel that the inpatient setting leaves open more treatment options and quicker recovery.

Our hypothesis is that using evidence-based criteria, DM patients can be managed appropriately in the outpatient setting. This study was set up to track the clinical outcomes, identify relevant factors, and describe treatment patterns that optimize hand infection resolution in the DM patient, specifically managed in the outpatient setting.

## Materials and methods

Patients were managed on an outpatient basis. This did not include patients kept in an observation unit. Patients were seen in the emergency department, the management plan was developed, and interventions performed, and the patients were discharged for follow-up in the clinic. Management criteria were developed from clinical experience and evidence-based criteria. The criteria included the following: chronic or recurrent infection, concern regarding the patients’ ability to comply with instructions, evidence or high suspicion for deep palmar space infection, evidence or high suspicion of pyogenic (suppurative) flexor tenosynovitis, failure of outpatient management, immunocompromised patient, open fracture, previous multiple hand infections (< 3), polytrauma, and systemic signs of infection [14-18]. Briefly, patients at risk for systemic infection, history of immunocompromise, previous hand infections (> 3 episodes), or those with other reasons to be hospitalized were excluded from outpatient management. Study exclusion criteria included age < 18 years old, post-surgical infections, patients treated in the operating room, inpatient admission (> 24 hours) for intravenous (IV) antibiotics (abx), and those with incomplete data. All cases were tracked until resolution for infection as identified in the electronic health record.

Treatments were divided into bedside interventions, IV abx, and oral antibiotics (po abx). Bedside interventions included both incisional and excisional (debridement) procedures. All outpatient procedures were performed wide awake with local anesthesia and no tourniquet. Both IV and po abx therapies were analyzed based on an intent to treat model. Patients were identified as DM if the hemoglobin A1c was greater than 6.5% or already diagnosed with DM.

The primary measured outcome was the resolution of the hand infection at two weeks and two months from the initial presentation. Also, unplanned return to the emergency department and conversion to inpatient care were tracked as proxies for the failure of the treatment plan. Secondary outcomes included the response to specific pathways, as well as the demographic and patient characteristic comparisons of the different treatment groups (DM versus non-DM and outpatient versus inpatient).

**Figure 1:**
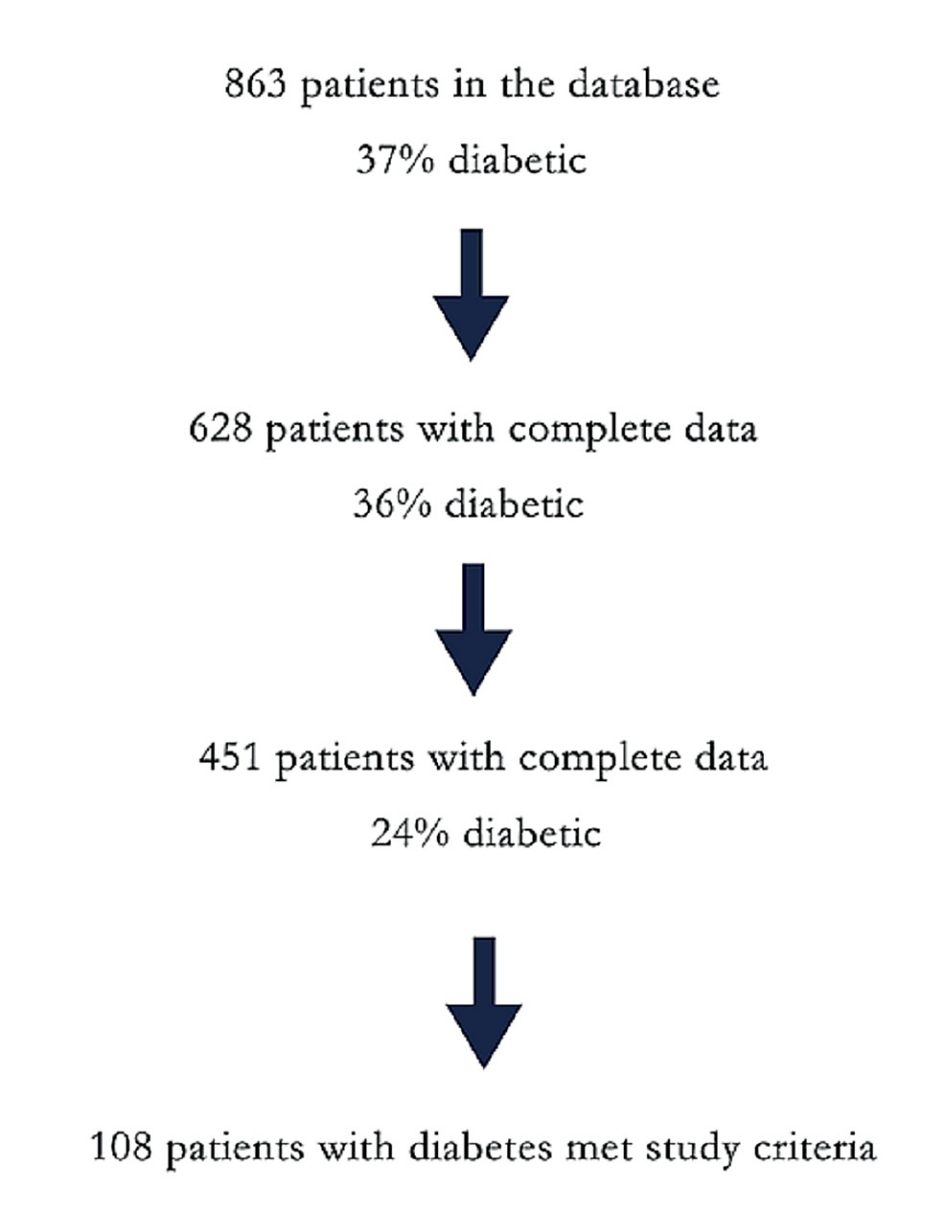
Database cases meeting inclusion criteria

For the study interval, January 2013 to December 2018, there were 628 patients in the database with complete data treated in both inpatient and outpatient settings. Of that group, 451 patients were treated as outpatients and met the inclusion criteria. One-hundred-eight patients (24%) were identified with DM (Figure 1).

The patients were sub-grouped by the treatments performed. All patients were placed in one of six groups based on a combination of IV abx, po abx, and interventional procedure (pro) (Table 1). Group A received IV abx, bedside intervention, and po abx (+IV abx, +pro, +po abx). Group B received IV abx as well as po abx but did not undergo bedside intervention (+IV abx, -pro, +po abx). Group C received IV abx and underwent a bedside intervention but did not get po abx (+IV abx, +pro, -po abx). Group D received IV abx but did not undergo bedside intervention nor did they receive po abx (+IV abx, -pro, -po abx). Group E only received po abx with no IV abx and no bedside intervention performed (-IV abx, -pro, +po abx). Group F did not receive IV or po abx and did not undergo a bedside intervention (-IV abx, -pro, -po abx). The institutional review board approved the study and guidelines were followed.

**Table 1:** Outpatient treatment subgroups with percentage improvement at two weeks and two months after treatment DM, diabetes mellitus; IV abx, intravenous antibiotics; po abx, oral antibiotics; pro, bedside procedure * One patient with delayed diagnosis of osteomyelitis of the distal phalanx. Required six weeks of oral antibiotics beginning at two point five (2.5) months after initial presentation. Complete resolution at four months.

Group	Treatment	Patients (n)	Resolution at 2 Weeks (%)	Resolution at 2 Months (%)	Unplanned Return to the Emergency Department (n)	Converted to Inpatient Care (n)^*^
A	+IV abx, +pro, +po abx	29	26 (90)	29 (100)	2	0
B	+IV abx, -pro, +po abx	13	7 (57)	13 (100)	1	2
C	+IV abx, +pro, -po abx	10	7 (70)	10 (100)	0	1
D	+IV abx, -pro, -po abx	19	6 (32)	19 (100)	0	4
E	-IV ABX, -pro, +po abx	28	20 (72)	27 (95^*^)	0	5
F	-IV ABX, -pro, -po abx	9	3 (37)	9 (100)	0	1
	Total DM	108	67 (62)	107 (99*)	3	10
	Total Non-DM	343	257 (75)	343 (100)	6	24

Statistical analysis

Statistical analysis was performed using a two-sample Wilcoxon test to compare healing for different characteristics and categories. Data were analyzed using Fisher’s exact test and the chi-square test.

## Results

For non-DM patients (n = 343) managed in the outpatient setting, 257 (75%) achieved resolution of infection at 2 weeks and 343 (100%) at 2 months. For the outpatient DM patients (n = 108), at 2 weeks 67 (62%) and at 2 months 107 (99%) achieved resolution of infection. The difference between the two groups was statistically significant at two weeks (p = 0.024) but not at two months. One DM patient was treated for distal phalanx osteomyelitis, which resolved at four months. In comparing the two populations, we were not able to discern any confounding factors with respect to demographics or types of interventions. Wound cultures were performed for 49 (45%) of the DM patients versus 127 (37%) for the non-DM patients among the outpatient groups. Wound culture profiles were not substantially distinctive for the DM and non-DM groups. Similar percentages for methicillin-resistant and non-resistant Staphylococcus aureus were seen in both groups (Table 2). Polymicrobial culture results were also equivalent for the two groups. There were no reported complications from the interventions or antibiotic-related adverse reactions. There were no amputations or deaths in either outpatient arm.

**Table 2:** Comparison of hand infection patients DM, diabetes mellitus; HgA1c, hemoglobin A1c; MRSA, methicillin-resistance Staphylococcus aureus; po abx, oral antibiotics; IV, intravenous *Significant comorbidities, including hypertension, myocardial infarction or cerebrovascular accident, acquired immunodeficiency, transplant, and Raynaud’s disease †Wound cultures were only measured for patients undergoing an incisional or excisional procedure or who had an open wound ‡Baseline for this is the number of cultures performed, not the number of patients in the group §Includes patients with previous history or current documentation of intravenous drug abuse ‖Includes patients lost to follow-up

	Outpatient DM (n=108)	Outpatient non-DM (n=343)	Inpatient DM (n=64)	Inpatient non-DM (n=113)
Patients, n	108	343	64	113
Age, years	47 + 25	48 + 33	52 + 31	50 + 27
Gender, male (%)	72 (67)	213 (62)	39 (61)	67 (59)
HgA1c, %	8.7	-	9.2	-
Other comorbidities, (%)^*^	13 (12)	34 (10)	46 (72)	85 (75)
Wound culture performed, (%)^†^	49 (45)	127 (37)	61 (95)	108 (96)
MRSA culture^‡^	27	25	37	24
Polymicrobial culture^‡^	36	32	47	51
Gram-negative or anaerobic culture^‡^	4	0	3	2
Received IV abx, (%)	71 (66)	250 (73)	64 (100)	113 (100)
Received po abx	57	52	100	96
Imaging performed	68	61	87	83
Incisional or excisional procedure	38	29	100	94
Intravenous drug abuse^§^	6	14	8	20
Worker compensation case	0	1	0	0
Converted to inpatient care (%)	10 (9)	24 (7)		
Resolution at 2 months (%)	107 (99)	343 (100)	64 (100)	113 (100)
Incomplete data^‖^	21	30	24	28

We reviewed the different treatment pathways among the outpatient DM patients with respect to recovery at two weeks. The highest percentage of recovery was Group A (+IV abx, +pro, +po abx; n = 29) at 26 (90%) improved (Table 1). That was followed by patients who only received po abx (n = 28), 20 (72%). The next highest recovery group was Group C (n = 10), which did not receive po abx but did receive both IV abx and a procedure, with seven (70%) resolutions at two weeks. Group E had the highest outpatient failures, with five patients converting to inpatient. The lowest recovery rates at two weeks were noted for the other three groups that did not receive a procedure (Groups B, D, and F).

Conversion to inpatient care was similar for the DM and non-DM groups at 9% (10) and 7% (24), respectively. Unplanned return to the emergency department was equivalent for the outpatient managed DM patients with three (3%) and non-DM patients with seven (2%).

We compared the results of inpatient to outpatient care during the study interval. Overall recovery at two months was equivalent (Table 2). The inpatient group (n = 177) had a much higher proportion of comorbidities than the outpatient group (n = 451), with 131 (74%) versus 47 (11%), respectively. Inpatients had a higher percentage of wound cultures performed 169 (96%) versus 176 in the outpatients' group (39%). While 100% of inpatients received IV abx, only 70% (316) of outpatients did. Similarly, 97% (172) of inpatients were discharged with po abx, and only 59% (267) of outpatients received it. Almost all (168, 95%) of the inpatients underwent a procedure while only 32% (144) of outpatients did. Incomplete data, resulting in exclusion from this comparison, was 28% in the outpatient group and 26% for the inpatients.

## Discussion

In the 108 DM patients, we saw resolution for 62% (67) of outpatient infections at two weeks and 99% (107) at two months. For the non-DM cohort (343), there was 75% (257) and 100% (343) resolution, respectively, for the same time points. The statistical difference present at the two-week interval was absent at two months. Conversion to inpatient care was less than 10% for both groups. Our criteria appear to allow for safe outpatient management of hand infections, including patients with DM. Our results suggest that DM may not necessarily be a risk factor in hand infections that portend a poor outcome.

Although this is an observational study, some interesting findings emerged from the treatment pathways. Both po and IV abx are indicated for cellulitis and clinical signs of infection. Incisional and excisional debridements are indicated for closed space infections and at-risk tissue. Patients who received procedural and antimicrobial treatments had the best outcomes. These patients had the nidus of the infection removed by the procedure, allowing the abx to work successfully. The next highest success was for patients only receiving po abx. This group presumably had an early stage cellulitis and responded appropriately. Closely behind this group were patients who received a procedure and IV abx, but no po abx. This also suggests that the procedure removed the nidus of infection, and the need for prolonged abx was limited. The slowest rate of recovery was in the group that only received a single dose of IV abx. This group also had the highest conversion to inpatient care (17%). This group represents a high risk for treatment failure. If the goal of abx care is the treatment for cellulitis, one IV dose may not be the appropriate length of treatment. Further review of this group suggests that the ones that do better may have been misdiagnosed. They may have been superficial burns and inflammatory processes. Group F, which received no treatment, had a similar slow resolution. We believe this group also included many misdiagnosed patients.

We compared our outpatient management with inpatient management of hand infection during the same interval (Table 2). Patient profiles with respect to age, gender, and history of IV drug abuse were not dissimilar between the groups. The percentage of patients with incomplete data for all groups ranged from 21%-30%. The inpatients all received IV and almost uniformly received po abx at discharge. Inpatients were more likely to undergo a procedure and diagnostic imaging. Not surprisingly, the inpatients had more comorbidities, which may explain the need for close monitoring and more involved treatment protocols.

Our findings compare favorably with other studies. Gonzalez et al. showed that many hand infections (40%) can be successfully treated with a single procedure and appropriate abx [11]. Sharma et al. treated 35% of DM hand infections with outpatient incision and drainage [19]. Koshy and Bell concur that most hand infections can be managed in an outpatient setting [20]. Our percentage of DM patients is included in Table 1. In our study, 42% of DM patients were managed with the outpatient guidelines successfully, in line with the above-cited studies.

There are limitations of this study. The patients who were lost to follow-up averaged 26%. These patients could represent patients who went elsewhere for definitive care. As an observational study, the treatment pathways cannot be compared head-to-head. We believe the methodology is sound and transparent. We applied the guidelines for triaging outpatient care and reviewed the outcomes. The low conversion to inpatient care reinforces the role of outpatient care even with DM patients. Diabetes was managed based on the treatment regimen already developed for the patients. No patients were newly diagnosed. We did not track adherence to the treatment plan or serum blood glucose. We also did not analyze results between those managed with insulin versus oral hypoglycemic agents. DM does not seem to be an independent risk factor for poor outcomes in the outpatient setting.

## Conclusions

We have shown that the management of hand infections for DM patients can be safely and effectively performed in the outpatient setting. When indicated, IV abx and bedside intervention resulted in the highest probability of success at two weeks. Hand specialists must remain vigilant and engaged in the management of these patients to improve recovery and limit recidivism.
